# SHKBP1 is a target for sepsis: evidence from WGCNA and multiple machine learning algorithms

**DOI:** 10.3389/fimmu.2025.1709188

**Published:** 2025-12-08

**Authors:** Fan Wu, Junlin Lu, Yi Liu, Runquan Zhou, Wenjuan Li, Mingxing Wang, Shan Xu, Yuanhui Sheng, Dan Zhang

**Affiliations:** 1 Department of Emergency Medicine, The First Affiliated Hospital of Chongqing Medical University, Chongqing, China; 2 Department of Emergency Medicine, The Second Affiliated Hospital of Chongqing Medical University, Chongqing, China

**Keywords:** sepsis, SHKBP1, cuproptosis-related genes (CRGs), machine learning, weighted gene co-expression network analysis (WGCNA)

## Abstract

**Background:**

Identifying novel biomarkers for sepsis is essential for improving patient outcomes. Cuproptosis, a recently discovered form of cell death associated with various diseases, has an unclear relationship with sepsis. This study aimed to elucidate the expression patterns of cuproptosis-related genes(CRGs) in sepsis, identifying potential biomarkers and therapeutic targets.

**Methods:**

We investigated the expression patterns of cuproptosis-related genes in sepsis and performed consensus clustering. A diagnostic model for sepsis was constructed using weighted gene co-expression network analysis (WGCNA) combined with four machine learning algorithms. Prognosis-related genes were identified via Kaplan-Meier survival analysis and validated in septic mice.

**Results:**

We identified 28 differentially expressed CRGs and characterized a specific immune landscape. Our findings showed that sepsis samples could be divided into two clusters based on CRGs expression. We established a diagnostic model based on five key genes(SHKBP1,ICAM2,CTSD,SNX3, and SLC22A4), and Kaplan-Meier survival analysis revealed that SHKBP1 was significantly correlated with both the diagnosis and prognosis of sepsis.

**Conclusion:**

Our study provides a comprehensive analysis of CRGs expression in sepsis, establishes a diagnostic model, and identifies SHKBP1 as a biomarker for both diagnosis and prognosis prediction, offering new insights for sepsis management.

## Introduction

Sepsis is a systemic inflammatory response triggered by infection, characterized by rapid progression and complex mechanisms, often accompanied by excessive immune system activation, leading to multiple organ dysfunction syndrome (1, 2). Sepsis affects millions of individuals worldwide each year, and mortality rates remain high. Among critically ill patients, mortality rates can reach 30% to 50% (3–5). Existing treatments—including antibiotics, anti-inflammatory drugs, and fluid resuscitation—can improve the clinical condition in sepsis but often fail to significantly reduce mortality (6, 7). The pathological process of sepsis involves complex interactions including inflammatory responses, oxidative stress, increased vascular permeability, and metabolic disorders (8–11). Therefore, current research is increasingly focusing on the discovery of early diagnostic biomarkers and the development of new therapeutic targets, aiming to improve patient survival rates through earlier and more precise interventions (12, 13).

In recent years, cuproptosis, a novel mechanism of cell death, has attracted increasing attention from sepsis researchers (14). Cuproptosis is a form of cell death first proposed by Tsvetkov et al. in 2022 (15). Its mechanism involves intracellular excess copper ions binding to lipoylated proteins of the tricarboxylic acid cycle, leading to protein aggregation and depletion of iron-sulfur cluster proteins. This induces mitochondrial dysfunction and excessive generation of reactive oxygen species, ultimately causing cellular toxicity (15). Current studies have shown that cuproptosis is associated with various diseases, including cancer, Alzheimer’s disease, and cardiovascular diseases (16–18), and plays an important role in regulating immune responses and inflammatory pathways (19). Given that the pathological process of sepsis also involves immune dysregulation and inflammatory responses (9), it is speculated that cuproptosis may participate in the onset and development of sepsis. However, the specific mechanisms of cuproptosis in sepsis remain unclear. As research progresses, strategies to regulate cuproptosis may bring new insights into disease treatment, providing a novel approach to improve conditions by modulating copper metabolism (20). Therefore, exploring the role of cuproptosis in the pathogenesis of sepsis and unveiling the expression patterns of cuproptosis-related genes(CRGs) in sepsis will contribute to developing new therapeutic targets for sepsis.

In this study, we aimed to comprehensively characterize the expression patterns of CRGs in sepsis and to elucidate their potential diagnostic and prognostic value. Using multiple publicly available transcriptomic datasets, we identified differentially expressed CRGs, explored sepsis molecular subtypes through consensus clustering, and constructed a diagnostic model by integrating weighted gene co-expression network analysis (WGCNA) with several machine-learning algorithms. Furthermore, we validated the key findings using survival analysis and experimental verification in a murine cecal ligation and puncture (CLP) model. This integrative strategy provides a framework for understanding the potential role of cuproptosis in sepsis and may help identify novel molecular targets for clinical translation.

## Materials and methods

### Data processing

Seven datasets were obtained from the Gene Expression Omnibus (GEO) database(https://www.ncbi.nlm.nih.gov/geo/) using R software(version 4.3.1), with detailed datasets information shown in **Table 1**. The GSE65282 RNA-seq dataset was used to develop a diagnostic model for sepsis. This dataset includes 802 whole blood samples, consisting of 42 healthy controls and 760 sepsis samples, among which 479 sepsis samples have available clinical prognosis data. The remaining six datasets include a total of 190 normal controls and 503 sepsis samples and were used to validate the stability of the model. The “SVA” R package(v3.50.0) was used to eliminate batch effects and normalize the data. All datasets are publicly available.

**Table 1:** Key characteristics on the datasets included in the study.

Dataset	Platform	Sample species	Sample organism	Healthy control	Sepsis
GSE65282	GPL15870	Homo sapiens	Whole blood	42	760
GSE8121	GPL570	Homo sapiens	Whole blood	15	30
GSE13904	GPL570	Homo sapiens	Whole blood	18	158
GSE26440	GPL570	Homo sapiens	Whole blood	32	98
GSE28750	GPL570	Homo sapiens	Whole blood	20	10
GSE95233	GPL570	Homo sapiens	Whole blood	22	51
GSE134347	GPL17586	Homo sapiens	Whole blood	83	156

### Acquisition, difference analysis, expression correlation analysis, and immune cell infiltration analysis of CRGs

We obtained 48 genes related to “cuproptosis” from the GeneCards database(https://www.genecards.org/).The “limma”(v3.58.1) R package was used to identify differentially expressed genes(DEGs), while “pheatmap” and “ggpubr” R packages were employed for visualizing the results. The correlation between DEGs and their visualization were performed using the “corrplot”(v0.95) and “circlize”(v0.4.16) R packages. Subsequently, the “CIBERSORT”(v1.03) R package was used to analyze immune cell infiltration between sepsis samples and normal control samples, and further analysis was conducted on the correlation between DEGs and immune cells infiltration.

### Consensus clustering based on CRGs

Based on the expression of CRGs, the “ConsensusClusterPlus” R package(v1.66.0) was used for consensus clustering of the GSE65682 dataset to better distinguish sepsis subtypes. The number of clusters ranged from 2 to 9, and the process was repeated 50 times to ensure stability, with 80% of the total samples randomly extracted for each time. The clustering algorithm was set to “k-means” (clusterAlg = “km”), and the Euclidean distance (distance = “Euclidean”) was used as the distance metric. Principal component analysis (PCA) was employed to visualize the clustering results.

### Biological function and pathway enrichment analysis

The Gene set variation analysis(GSVA)(v1.50.5) package was used for enrichment analysis of CRGs in terms of cellular components, molecular functions, biological processes, and pathways. The “c2.cp.kegg.symbols.gmt” and “c5.go.symbols” gene sets were obtained from the Molecular Signatures Database (MSigDB).Gene Ontology(GO) and Kyoto Encyclopedia of Genes and Genomes(KEGG) enrichment analyses were conducted using the “clusterProfiler”(v4.10.1) package. Significant terms were showed in a barplot and a p-value <0.05 considered a statistically significant term.

### Identification of hub genes using WGCNA

First, the top 25% of genes with the highest variation were selected for WGCNA (v1.73)analysis. Pearson correlation matrices and average linkage methods were used to construct a similarity matrix. Next, a weighted adjacency matrix was generated using a soft-thresholding parameter, which was subsequently transformed into a topological overlap matrix (TOM). Finally, hierarchical clustering based on TOM dissimilarity was performed, with a minimum gene module size of 100.GO enrichment analysis was conducted to explore the functions of hub genes with gene significance (GS) > 0.2 and module membership (MM) > 0.8.

### Intersection genes and establishment diagnostic model for sepsis using multiple machine learning algorithms

The “VennDiagram” R package(v3.50.0) was used to obtain intersection genes by intersecting the hub genes identified through WGCNA. These intersection genes were utilized for subsequent analysis using machine learning algorithms. We formulated the analysis as a binary classification task to distinguish sepsis from healthy controls based on the expression profiles of cuproptosis-related genes. Four machine learning algorithms—Random Forest (RF), Support Vector Machine (SVM), Extreme Gradient Boosting (XGB), and Generalized Linear Model (GLM). The machine learning models were supported by the “caret” R package(v6.0.94) and constructed using the “kernlab”(v0.9.33), “randomForest”(v4.7.1.2), and “XGBoost”(v1.7.8.1) R packages. Models were trained with repeated stratified 5-fold cross-validation (trainControl with classProbs=TRUE, summaryFunction=twoClassSummary, and metric=“ROC”), using AUC as the primary evaluation metric. To improve model robustness and prevent overfitting, lightweight hyperparameter tuning was performed with predefined parameter grids: RF (mtry = 2, 3, 5), SVM (C = 0.5, 1, 2), XGB (max_depth = 4, 6; eta = 0.3, 0.1), and GLMnet (lambda = 0.001–1). Up-sampling was applied during resampling to handle class imbalance. Model performance was evaluated on the testing set using ROC/AUC. We further explored the models using the explain function of the “DALEX”(v2.4.3) R package, and RMSE from DALEX was used as a supplementary indicator of prediction stability. We divided the dataset into training and validation sets with a 7:3 ratio and then proceeded with model construction. Key parameters included “5-fold cross-validation” for RF, “svmRadial” for the SVM, and “xgbDART” for XGB. The “pROC”(v1.18.5) R package was used to evaluate the predictive performance of the models. Finally, we used the “rms”(v6.8.1) R package to construct a nomogram and the “rmda”(v1.6) R package to create decision curves and calibration plots to evaluate the model’s performance and decision-making effectiveness. A schematic flowchart illustrating the complete modeling process—from data preprocessing to model validation—is provided in **Supplementary Figure S5**.

### Animal experiments

C57BL/6 mice (6–8 weeks old) were obtained from the Animal Experiment Center of Chongqing Medical University. The sepsis model was established using CLP. Mice were anesthetized with intraperitoneal injection of pentobarbital sodium (50 mg/kg), and a 1-1.5 cm incision was made along the midline of the abdomen to expose the cecum. In the CLP group(n=6), a 4–0 suture was used to ligate the distal end of the cecum approximately 1 cm long, and a 20G needle was used to puncture the cecum, followed by gently squeezing out a small amount of feces before closing the abdomen. The sham-operated group(n=6) underwent all procedures except for cecal ligation and puncture.

### Histopathological analysis and quantitative real-time polymerase chain reaction

Lung tissue was fixed in 4% paraformaldehyde and stained with hematoxylin and eosin (HE).Total RNA was extracted using TRIzol reagent and reverse transcribed using the PrimeScript™ RT kit. Samples were prepared according to the Takara GreenmixTagII kit and detected by real-time quantitative PCR. Results were analyzed using the 2^-ΔΔCt^ method, with cDNA expression levels normalized to GAPDH.

### Statistical analysis

All data processing and statistical analysis were performed using R software (version 4.3.1) and GraphPad Prism software (version 9.0). Differences between groups were assessed using either the independent samples t-test or Mann-Whitney U test. K-M survival analysis was used to evaluate the relationship between gene expression and patients survival. A significance level of P < 0.05 was used.

## Results

### Identification of different expression patterns of CRGs and immune cell infiltration analysis

**Figure 1:**
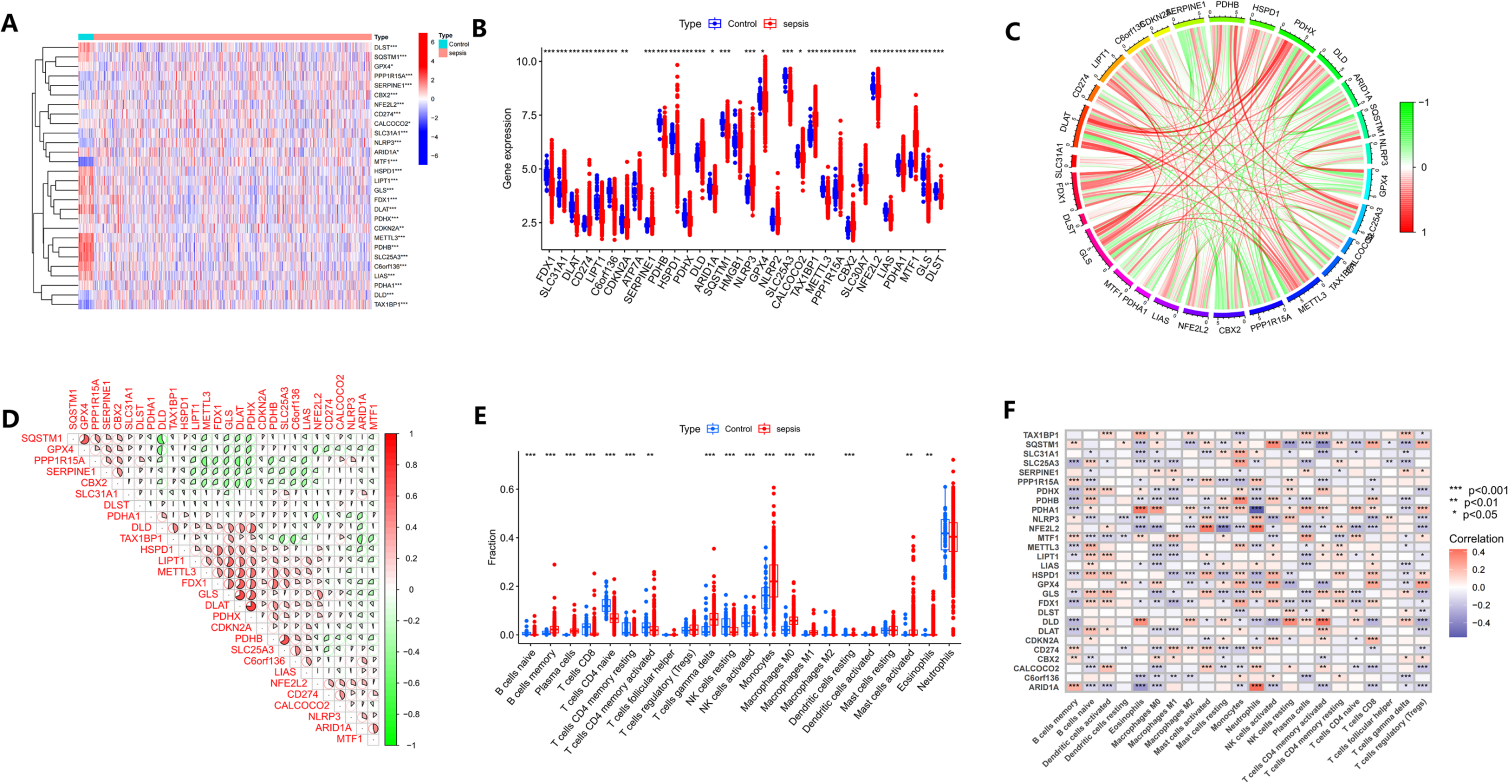
Expression patterns of CRGs and immune cell infiltration in sepsis and healthy controls. **(A, B)** Heatmap and boxplot showing the differential expression of 28 CRGs between sepsis and control samples. **(C)** Circos plot illustrating pairwise correlations (red = positive; green = negative). **(D)** correlation matrix visualizing the strength and direction of these associations. **(E)** Estimated relative proportions of 22 immune cell types in sepsis and control groups, calculated using the CIBERSORT algorithm and displayed as stacked bar plots. **(F)** Spearman correlation heatmap demonstrating the associations between CRG expression and immune cell infiltration levels.*p < 0.05; **p < 0.01; ***p < 0.001.

To investigate the expression of CRGs in sepsis and their relationship with immune cells, we extracted the expression data of CRGs from the GSE65282 dataset, which includes 42 normal controls and 760 sepsis samples. A total of 28 DEGs were identified, with 10 genes upregulated and 18 downregulated. We visualized the results of the DEGs using barplot (**Figure 1A**) and heatmaps (**Figure 1B**). Correlation analysis indicated potential regulatory relationships among these genes (**Figures 1C, D**). Using the CIBERSORT algorithm, we estimated the immune cell abundance in the GSE65282 dataset (**Figure 1E**) and analyzed the correlation between DEGs and immune cell abundance (**Figure 1F**). The results showed a significant correlation between CRGs and immune cells, particularly monocytes and naive CD4^+^ T cells. In summary, our findings revealed the CRGs were potentially associated with sepsis-related immune and inflammatory pathways.

### Unsupervised clustering analysis of sepsis samples

**Figure 2:**
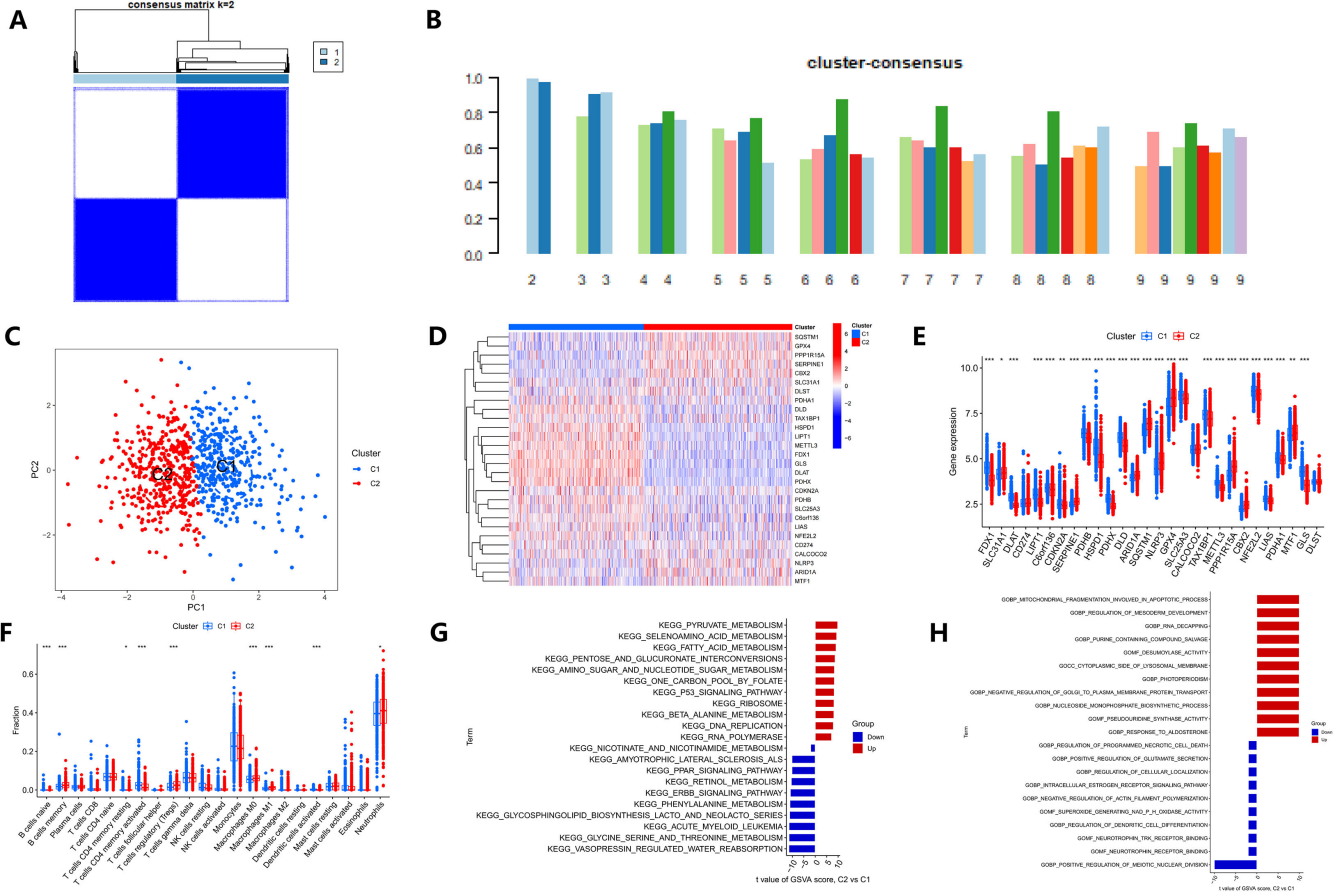
Consensus clustering and functional characterization of sepsis subtypes based on CRGs expression. **(A)** Consensus matrix when k = 2, showing a stable partition of samples into two distinct clusters (C1 and C2). **(B)** Consensus score plots illustrating clustering stability across different k values. **(C)** PCA confirming clear separation between the two clusters. **(D, E)** Heatmap and boxplot displaying the differential expression patterns of CRGs between clusters C1 and C2. **(F)** Comparison of immune cell infiltration between clusters, estimated using the CIBERSORT algorithm; differences in immune cell proportions are indicated. **(G, H)** Gene Set Variation Analysis (GSVA) identifying differentially enriched pathways between clusters: **(G)** KEGG pathways and **(H)** GO biological processes. *p < 0.05, **p < 0.01, ***p < 0.001.

To determine whether distinct molecular subtypes of sepsis exist based on CRG expression, we performed consensus clustering analysis on 760 samples from the GSE65282 dataset. The consensus matrix indicated that two distinct clusters represented the optimal result (**Figure 2A**; **Supplementary Figures S1A–H**). The cumulative distribution function (CDF) curve, which measures the consensus stability of clustering results at different cluster numbers (k), was used to determine the optimal cluster count. The results showed minimal fluctuation and the best stability when k=2 (**Supplementary Figures S1I, J**). Moreover, when k=2, the consensus scores for each cluster were greater than 0.8 (**Figure 2B**). As a result, we divided the sepsis samples into two clusters, Cluster 1 and Cluster 2. The reliability of the clustering result was further confirmed by PCA analysis (**Figure 2C**).

To gain a more comprehensive understanding of the functional characteristics of the different clusters, we conducted further analysis. Among the 28 CRGs, 25 genes showed differential expression between the clusters (**Figure 2D**), with 15 genes downregulated and 10 genes upregulated in Cluster 2 compared to Cluster 1 (**Figure 2E**). Immune infiltration analysis using the CIBERSORT algorithm revealed significant differences in 9 immune cell types between the two clusters (**Figure 2F**). GSVA analysis indicated that C2 exhibited upregulation in pathways related to cell death, as well as metabolic pathways such as pyruvate and fatty acid metabolism, while C1 showed enrichment in pathways promoting nuclear division and metabolism of glycine, serine, and threonine (**Figures 2G, H**).

### WGCNA of DEGs in sepsis and clusters

**Figure 3:**
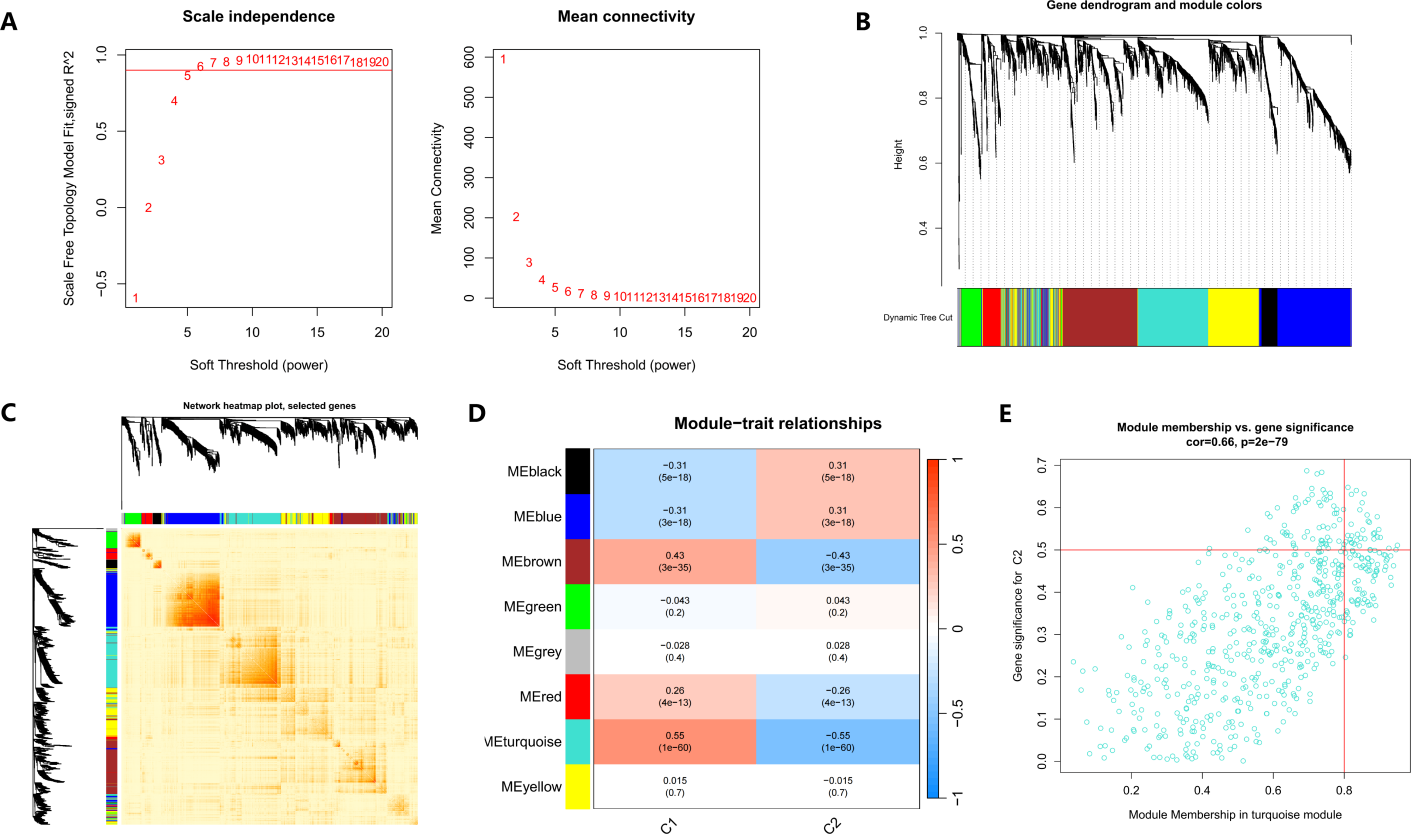
WGCNA analysis of two cuproptosis-related clusters. **(A)** Network topology analysis under different soft-thresholding powers for scale-free network construction. **(B)** Gene dendrogram and corresponding module colors representing co-expression clusters. **(C)** Heatmap of gene co-expression similarity among modules. **(D)** Correlation heatmap showing module–trait relationships between identified modules and the two clusters (C1 and C2). **(E)** Scatter plot illustrating the correlation between gene significance and module membership in the turquoise module (cor = 0.66, p = 2e−79).

To further explore the molecular heterogeneity between CRG-based clusters, we conducted WGCNA. A soft threshold of β = 6 was selected to ensure scale-free topology (**Figure 3A**). Hierarchical clustering identified distinct gene modules, each marked by different colors (**Figure 3B**), and the TOM heatmap further visualized gene clustering (**Figure 3C**). Eight modules were identified, with the “turquoise” modules highly correlating with Cluster 2 (cor = -0.55, p = 1e-60) (**Figure 3D**), from which a total of 566 hub genes were identified. Module membership in the turquoise module was significantly correlated with gene significance for Cluster 2 (cor = 0.66, p = 2e-79) (**Figure 3E**). These results highlighted distinct gene expression patterns across clusters.

To identify key gene modules associated with sepsis, we also performed WGCNA using the same workflow. First, we selected a soft threshold of β = 5, which ensured that the network followed a scale-free topology, as indicated by the scale independence and mean connectivity plots (**Supplementary Figure S2A**). Based on hierarchical clustering, gene modules were identified and represented by distinct colors (**Supplementary Figure S2B**). The TOM heatmap (**Supplementary Figure S2C**) demonstrated the clustering of genes with similar expression profiles. A total of eight modules were detected, with the “blue” module showing a strong correlation with sepsis (r = -0.58, p = 2e-73) (**Supplementary Figure S2D**). A total of 606 hub genes were subsequently extracted from this module for further analysis. Module membership in the blue module was also highly correlated with gene significance for sepsis (cor = 0.72, p = 1.5e-94), indicating that genes in this module were likely involved in sepsis-related transcriptional changes (**Supplementary Figure S2E**).

### Construction of sepsis diagnostic models using various machine learning algorithms

**Figure 4:**
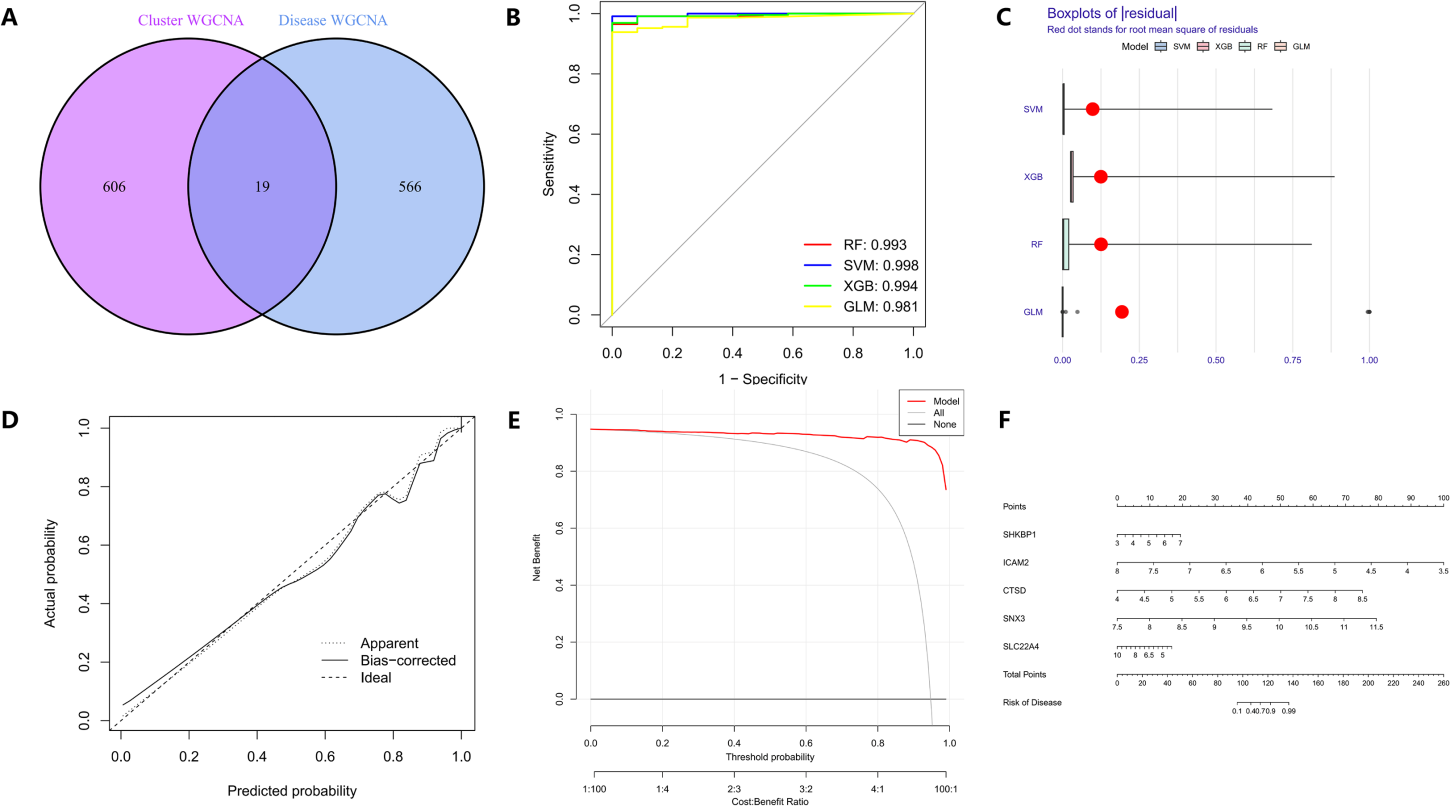
Construction and evaluation of the diagnostic model for sepsis using machine learning. **(A)** Venn diagram showing the intersection of hub genes identified by WGCNA from disease-related and cluster-related modules, yielding 19 common genes. **(B)** Receiver operating characteristic (ROC) curves comparing the diagnostic performance of four machine learning algorithms: RF, SVM, XGB and GLM. **(C)** Boxplots of residual distributions for the four algorithms, with red dots indicating root mean square residuals. **(D)** Calibration curve assessing the agreement between predicted and observed probabilities for the random forest model. **(E)** Decision curve analysis (DCA) demonstrating the clinical net benefit of the random forest model across different threshold probabilities. **(F)** Nomogram of the optimized random forest–based diagnostic model integrating the top five characteristic genes (SHKBP1, ICAM2, CTSD, SNX3, and SLC22A4) for individualized risk prediction of sepsis.

To establish a robust diagnostic model for sepsis and identify potential biomarker genes, we intersected the genes from WGCNA analysis of sepsis and cuproptosis clusters, resulting in 19 shared genes (**Figure 4A**). These genes were further screened using machine learning models (SVM, RF, XGB, and GLM). The receiver operating characteristic (ROC) curves indicated that the area under curve (AUC)of SVM, RF, and XGB models were greater than 0.99, suggesting excellent diagnostic performance (**Figure 4B**). The cumulative residual distribution maps and boxplots further validated the reliability of these models (**Figure 4C**, **Supplementary Figure S3B**). The top 10 genes with the lowest root mean square error (RMSE) in each model were presented in **Supplementary Figure S3A**. Nomograms for each model were then constructed, and based on calibration curves and decision curve analysis (DCA), the RF model was identified as the best diagnostic model. SHKBP1, ICAM2, CTSD, SNX3, and SLC22A4 were determined as hub genes (**Figures 4D–F**; **Supplementary Figures S3C–K**).

### Validation of the diagnostic model using external merged datasets and K-M survival analysis for prognostic gene identification

**Figure 5:**
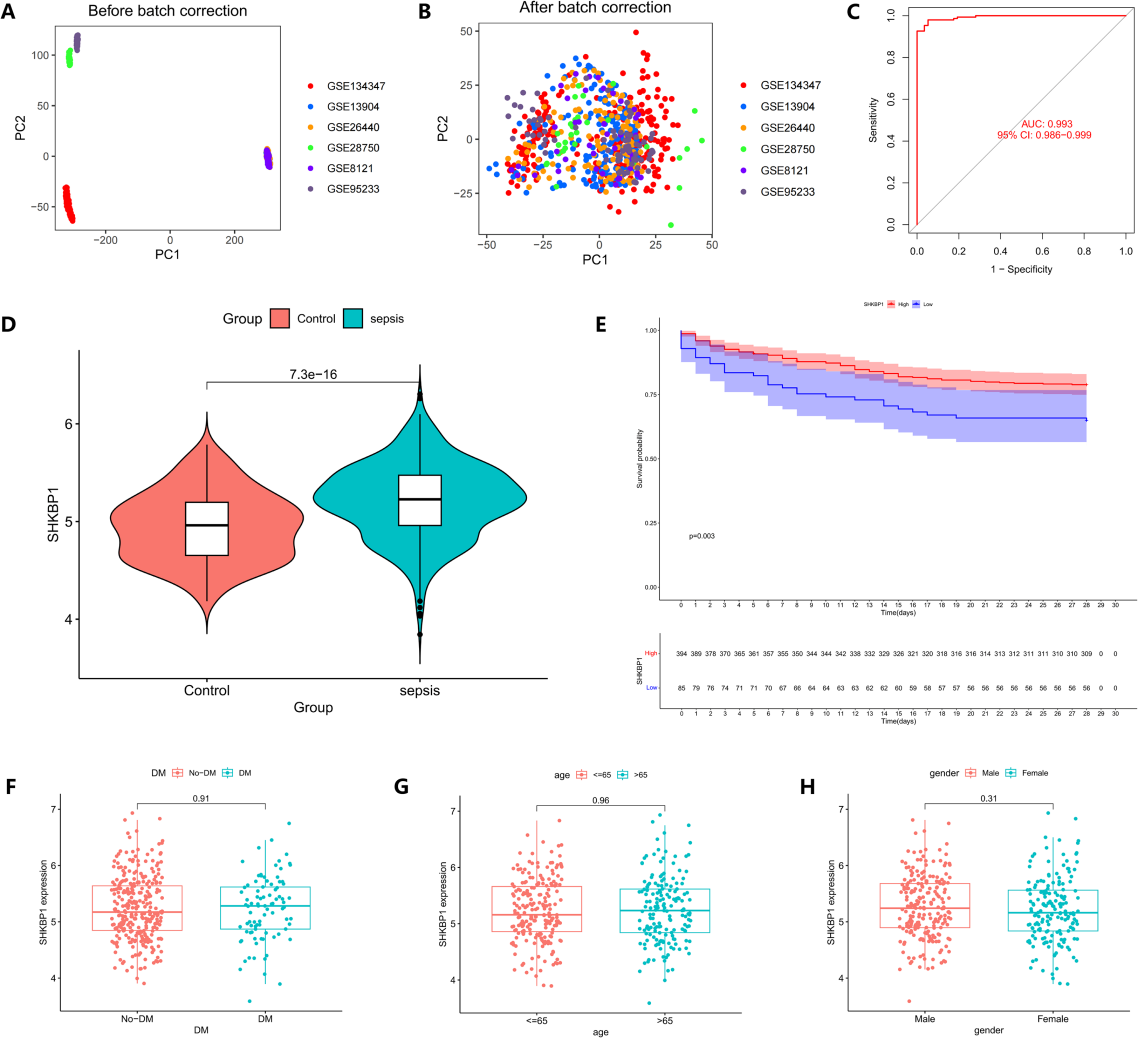
External validation and clinical correlation of the diagnostic model. **(A, B)** PCA plots before and after batch correction across six independent GEO datasets (GSE8121, GSE13904, GSE26440, GSE28750, GSE95233, and GSE134347), demonstrating effective removal of batch effects. **(C)** ROC curve showing excellent diagnostic performance of the random forest–based model in the validation cohort (AUC = 0.993, 95% CI = 0.988–0.999). **(D)** Violin plot displaying SHKBP1 expression levels in sepsis and control samples within the merged validation cohort. **(E)** Kaplan–Meier survival analysis of SHKBP1 expression in the GSE65282 dataset, indicating that higher SHKBP1 expression is associated with poorer survival outcomes. **(F–H)** Associations between SHKBP1 expression and clinical features, including diabetes mellitus **(F)**, age ≤ 65 vs. > 65 years **(G)**, and gender **(H)**.

To validate the generalizability of the diagnostic model, we merged six independent datasets to validate the diagnostic efficacy of the model constructed from the five identified hub genes. PCA analysis confirmed the successful elimination of batch effects (**Figures 5A, B**). The ROC curve demonstrated a high AUC value of 0.993 (**Figure 5C**), indicating strong diagnostic performance. Subsequently, we validated the expression levels of the five genes in the validation set, all of which showed significant differential expression (**Figure 5D**; **Supplementary Figures S4A–D**). To explore their potential prognostic relevance, K-M survival analysis revealed that only SHKBP1 was associated with sepsis prognosis (**Figure 5E**; **Supplementary Figures S4E–H**). Patients with high SHKBP1 expression had a significantly better prognosis than those with low expression (P = 0.003). To exclude the influence of factors such as age, sex, and diabetes status, we found no significant differences in SHKBP1 expression across these subgroups (**Figures 5F–H**). In summary, SHKBP1 was identified as a gene that could both diagnose sepsis and predict patient prognosis.

### Exploration of the molecular function of SHKBP1

**Figure 6:**
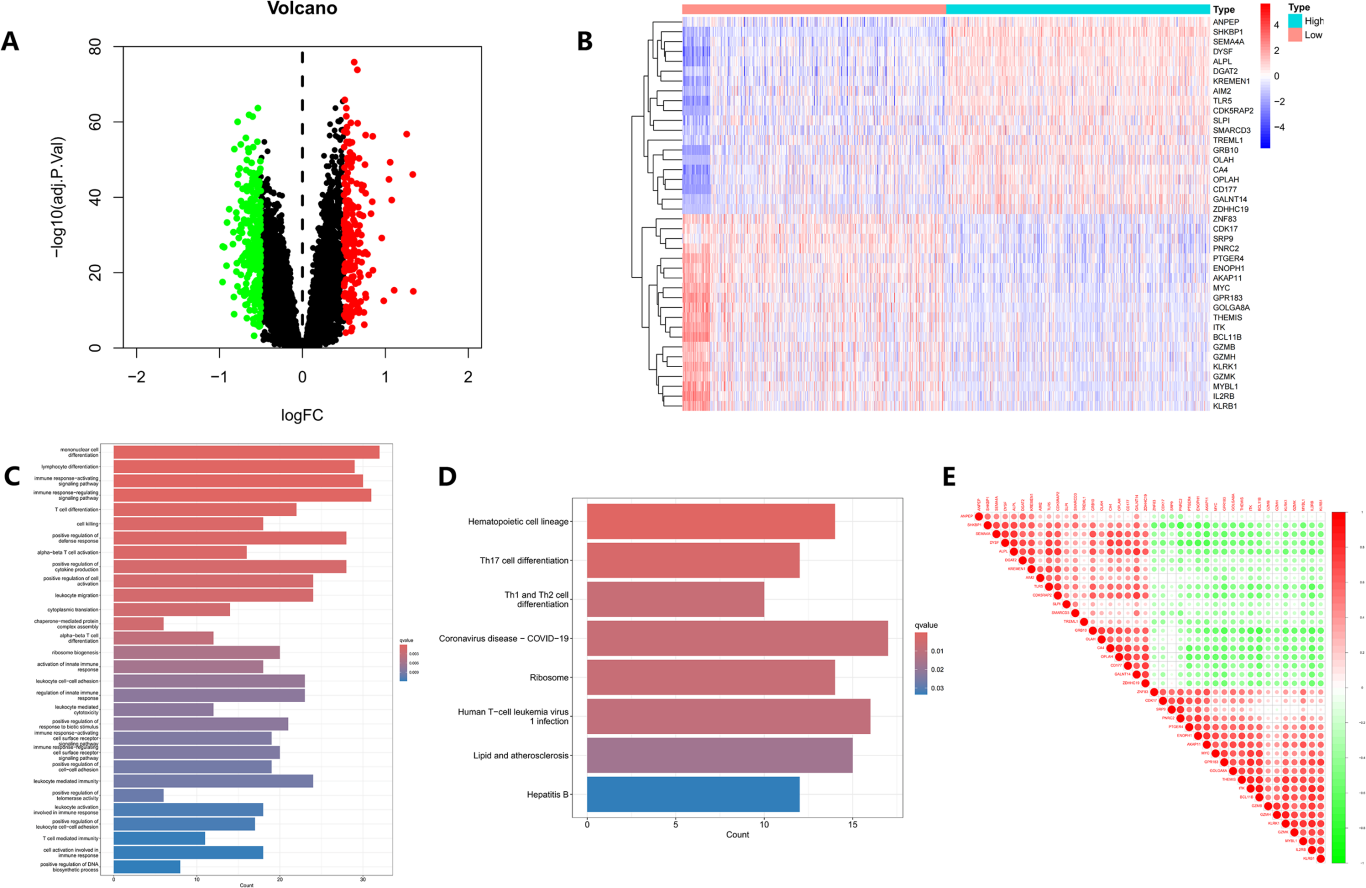
Molecular characteristics and functional enrichment analysis of SHKBP1. **(A, B)** Volcano plot and heatmap showing DEGs between high and low SHKBP1 expression subgroups. **(C, D)** GO and KEGG enrichment analyses of SHKBP1 related DEGs, mainly highlighting pathways associated with immune activation, cytokine regulation, and T-cell differentiation. **(E)** Correlation matrix illustrating the interrelationships among SHKBP1 related DEGs, with red and green representing positive and negative correlations respectively.

To clarify the potential biological roles of SHKBP1 in sepsis, we conducted further analysis on the GSE65282 dataset. The dataset was divided into two groups based on SHKBP1 expression levels, resulting in 251 downregulated and 210 upregulated genes (**Figure 6A**). A heatmap illustrated the expression patterns of these DEGs (**Figure 6B**). GO functional analysis suggested that the role of SHKBP1 is primarily associated with immune-related processes, such as activation of immune response, cytokine production, and the differentiation of monocytes, lymphocytes, and T cells (**Figure 6C**). KEGG pathway analysis indicated that SHKBP1 is involved in hematopoietic cell lineage and the differentiation of Th1, Th2, and Th17 cells (**Figure 6D**). Additionally, SHKBP1 showed interactions with multiple genes (**Figure 6E**). Our findings indicated a strong association between SHKBP1 and immune processes, suggesting that SHKBP1 might play a critical role in the pathogenesis and progression of sepsis.

### Validation of the expression of SHKBP1 in septic mice

**Figure 7:**
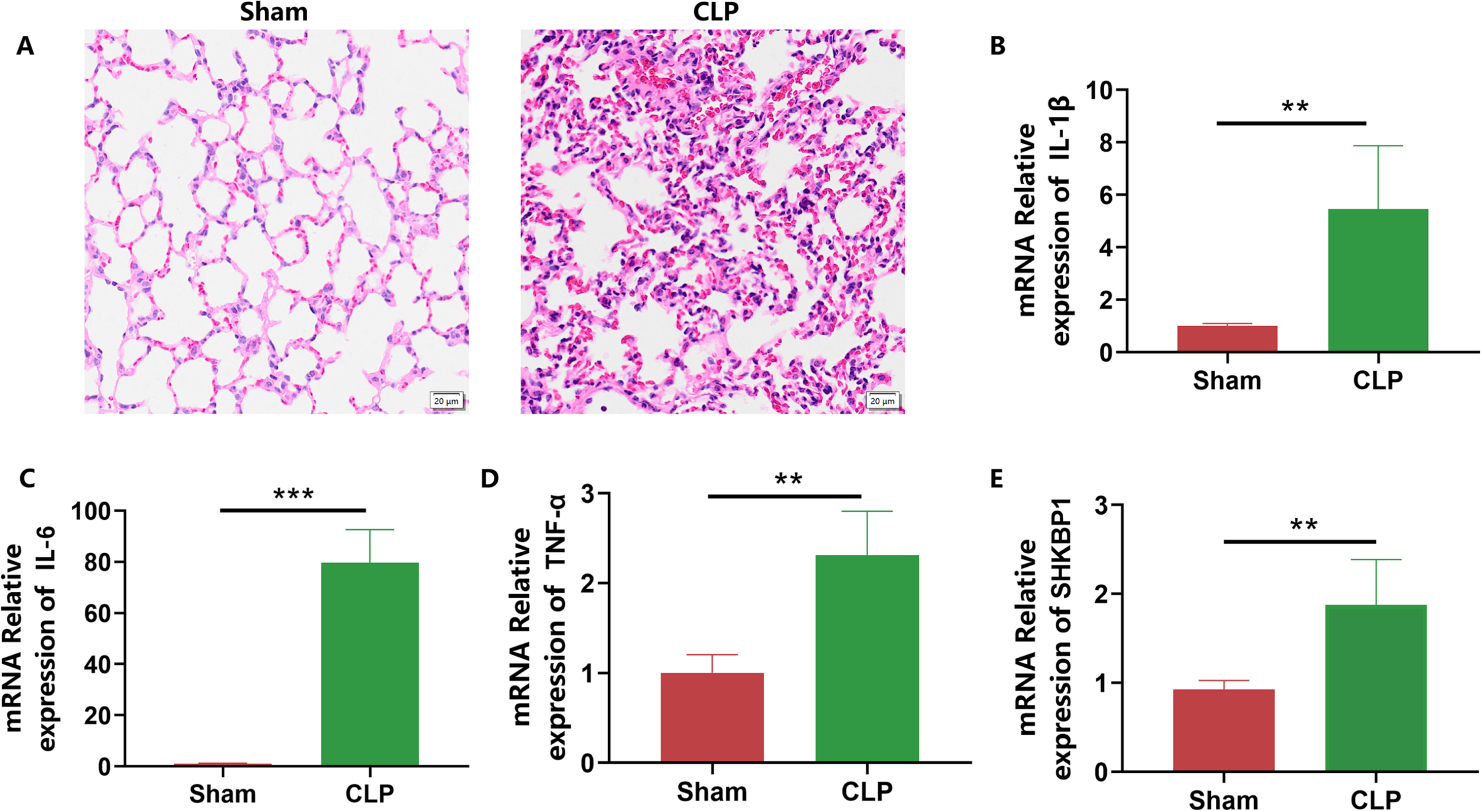
Validation of SHKBP1 expression in septic mice. **(A)** Representative H&E staining images of lung tissues from sham and CLP-induced septic mice, showing significant inflammatory infiltration and alveolar damage in the CLP group. **(B–D)** Quantitative RT–PCR analysis of inflammatory cytokines IL-1β, IL-6, and TNF-α in lung tissues, revealing markedly increased expression levels in septic mice compared to controls. **(E)** Relative mRNA expression of SHKBP1 in the lungs of septic mice, showing a consistent upregulation pattern with the bioinformatics findings. Data are presented as mean ± SD. **p < 0.01, ***p < 0.001.

To validate the expression of SHKBP1 during sepsis, we performed RT-qPCR on lung tissues from septic mice. Compared to the control group, septic mice exhibited significant lung damage (**Figure 7A**), with significant increases in inflammatory cytokines such as IL-1β, IL-6, and TNF-α in the lung tissues (**Figures 7B–D**). Furthermore, we observed a significant upregulation of SHKBP1 expression in the lung tissues (**Figure 7E**). These results provide *in vivo* evidence supporting SHKBP1 as a potential diagnostic and mechanistic biomarker in sepsis.

## Discussion

Sepsis is a disease with an extremely complex pathogenesis and persistently high mortality rate, making it one of the leading causes of death worldwide (5). Exploring effective biomarkers is crucial for achieving early diagnosis and precise treatment of sepsis, and holds the promise of improving patient prognosis (12). In this study, we constructed a sepsis diagnostic model based on the mechanism of cuproptosis, revealing that SHKBP1 is an important gene not only related to the early diagnosis of sepsis but also predictive of patient prognosis.

Cuproptosis is a newly discovered form of cell death, and its role in the pathogenesis of sepsis remains unclear. We analyzed the expression patterns of CRGs in patients with sepsis and identified 28 genes that were significantly differentially expressed, suggesting that cuproptosis may play a crucial role in the occurrence and development of sepsis. Macrophages, as frontline immune defenders, play a key role in the pathogenesis of sepsis (21). Our immune infiltration analysis showed a significant increase in the abundance of monocytes, M0 macrophages, and M1 macrophages in sepsis samples. The abundance of these cells was significantly positively correlated with CRGs such as GPX4 and PDHA1, and significantly negatively correlated with METTL3 and LIAS. Existing studies have shown that GPX4 is a negative regulator of ferroptosis (22), and activation of PDHA1 can ameliorate sepsis-induced acute kidney injury (23). Additionally, METTL3 has been proven to promote cell death, thereby exacerbating sepsis-induced acute lung injury (24). These findings further validate the relationship between gene regulation in our study and macrophage activity. Through consensus clustering analysis, we divided the sepsis samples into two distinct cuproptosis subtypes based on the expression patterns of CRGs, revealing two different biological processes. The CRGs and their immune cell infiltration characteristics differed between these subtypes, especially showing significant differences in the abundance of M0 and M1 macrophages. Furthermore, pathway enrichment analysis demonstrated significant differences in metabolic pathway enrichment—such as fatty acid metabolism—between the two subtypes. Studies have indicated that enhanced fatty acid metabolism is closely related to cuproptosis (25), and the relationship between cuproptosis and macrophages has also been supported by other research (26). Therefore, our study provides new insights into the interactions among cuproptosis, macrophages, and their metabolism.

In recent years, machine learning algorithms have made significant progress in sepsis research (27). By analyzing large amounts of patient data, machine learning can identify early symptoms and patterns of sepsis, enabling earlier diagnosis than traditional methods and significantly improving diagnostic accuracy through feature engineering and pattern recognition (28, 29). However, previous studies often suffered from small sample sizes, lack of validation cohorts, and reliance on single learning algorithms. To address these issues, we increased the sample size by merging multiple datasets and introduced a validation cohort, combining WGCNA with four machine learning algorithms for modeling. Ultimately, in our study, the RF algorithm achieved high accuracy in both the training and validation sets, with area under the curve (AUC) values exceeding 0.99, demonstrating the model’s stability and accuracy. Using this model, we identified five key genes for constructing the sepsis diagnostic model: SHKBP1, ICAM2, CTSD, SNX3, and SLC22A4. These genes also exhibited significant differential expression trends consistent with the training set in the validation set. Specifically, SHKBP1 is related to immune regulation and has been shown to be associated with tumor occurrence and metastasis (30). ICAM2 is an adhesion molecule that mediates interactions between leukocytes (such as lymphocytes and monocytes) and vascular endothelial cells, playing a key role in the rolling, adhesion, and transendothelial migration of immune cells, and is crucial for immune cell homing and inflammatory responses (31). CTSD encodes an aspartic protease located in lysosomes, participates in protein degradation, and plays an important role in antigen processing and presentation, aiding the immune system in recognizing and eliminating foreign pathogens (26). SNX3 is involved in membrane protein transport and cell signal transduction (32), while SLC22A4 encodes a transmembrane protein responsible for the transport of organic cations and carnitine and has been shown to be closely related to inflammation (33). Since these five genes are all closely related to inflammation and immune processes—and sepsis involves complex inflammatory and immune responses—the diagnostic model constructed based on these genes holds promise for the early and precise diagnosis of sepsis.

As the mortality rate of sepsis remains high, assessing its prognosis is a focal point of this study. Through K-M survival analysis, we found that among the five genes, only SHKBP1 was significantly correlated with sepsis prognosis (P = 0.003). SHKBP1 (SH3-domain kinase binding protein 1), also known as CIN85 (Cbl-interacting protein of 85 kDa), is a multifunctional adaptor protein containing SH3 domains. It plays a key role in various cellular processes such as signal transduction, endocytosis, cytoskeletal remodeling, cell migration, and immune regulation. As a multi-adaptor protein, SHKBP1 interacts with multiple signaling molecules to regulate intracellular signaling pathways. For example, it interacts with Src family kinases, Cbl, and other SH3 domain-containing proteins, affecting cell proliferation, differentiation, and survival (34). SHKBP1 plays an important role in the endocytosis and degradation of receptor tyrosine kinases (RTKs). It forms a complex with the E3 ubiquitin ligase Cbl, promoting the ubiquitination of RTKs and marking them for lysosomal degradation (35). Additionally, SHKBP1 interacts with multiple cytoskeleton-associated proteins through its SH3 domains, affecting the dynamic remodeling of actin, which is crucial for cell movement and morphology maintenance (36). SHKBP1 is also involved in immune regulation. Knocking out SHKBP1 can increase the number of CD8^+^ T cells (30); it is recruited to the T-cell receptor(TCR) signaling complex and mediates the inhibition of T-cell activation through binding with TCR signaling inhibitor-2, and it also affects B-cell activation (37). Moreover, SHKBP1 is associated with various diseases such as cervical cancer (38), ovarian cancer (39), lung cancer (40), and osteoarthritis (41). However, the relationship between SHKBP1 and sepsis remains unclear. We conducted preliminary validation in septic mice and found that the expression level of SHKBP1 in septic mice was higher than in the control group. Taken together, we have reason to believe that SHKBP1 is involved in the pathological mechanisms of sepsis and is related to its prognosis.

In this study, SHKBP1 was prioritized for *in vivo* validation because it exhibited the strongest association with clinical prognosis and immune-related pathways among the identified hub genes. Its consistent expression pattern across multiple datasets further supported its selection as a representative biomarker for experimental validation. Nevertheless, other hub genes such as ICAM1, CTSD, SNX3, and SLC22A4 also demonstrated biological relevance in sepsis and may contribute to disease pathogenesis through complementary mechanisms. Future studies incorporating additional molecular and cellular assays will be valuable to further validate and elucidate the roles of these candidate genes in sepsis progression.

Our study also has some limitations. First, our datasets were all derived from the publicly available GEO database, and the research mainly relies on bioinformatics analysis, lacking multicenter data validation. Therefore, although our findings indicate promising diagnostic potential, future prospective and multicenter studies are warranted to confirm the real-world diagnostic efficacy and translational applicability of the model. Second, we only conducted preliminary validation in a mouse model and require further animal experiments and clinical studies to confirm our findings. At last, The dataset used for model construction was imbalanced (479 sepsis vs. 42 controls). Although stratified sampling was applied to preserve class proportions and repeated cross validation was used to ensure stability, minor bias in model performance—particularly in AUC—cannot be fully excluded. Nevertheless, consistent trends across multiple metrics (AUC, accuracy, sensitivity, and specificity) and validation results suggest that the diagnostic models remain robust. Future studies based on larger, more balanced clinical cohorts will be valuable for further validation and optimization. In the future, we plan to conduct in-depth experimental investigations to delineate how SHKBP1 contributes to sepsis-induced immune dysregulation and its relationship with cuproptosis (particularly in macrophages and CD4^+^ T cells), and to carry out prospective clinical studies to evaluate the practical clinical utility of our diagnostic model.

## Conclusion

In conclusion, our study deeply explored the expression patterns of CRGs in sepsis, established a diagnostic model, and identified SHKBP1 as a biomarker that can be used for both diagnosis and prognosis prediction of sepsis. Our research provides new insights for the early diagnosis and precise treatment of sepsis.

## Data Availability

The original contributions presented in the study are included in the article/**Supplementary Material**. Further inquiries can be directed to the corresponding author.

## Supplementary material

Supplementary Figure 1 Consensus clustering analysis in sepsis samples. **(A–H)** Consensus matrices for different cluster numbers (k = 2–9), where the intensity of blue indicates the consensus within clusters. **(I)** Delta area plot showing the relative change in the area under the cumulative distribution function (CDF) curve for each k value. **(J)** CDF curves of consensus scores across different cluster numbers. **(K)** Histogram of cluster consensus scores, demonstrating optimal clustering stability when k=2.

Supplementary Figure 2 WGCNA of sepsis and healthy control samples. **(A)** Analysis of network topology across a range of soft-thresholding powers. The scale-free topology fit index (left) and mean connectivity (right) were used to determine the optimal power for network construction. **(B)** Gene dendrogram showing hierarchical clustering of genes into distinct co-expression modules, each represented by a different color. **(C)** Heatmap visualizing the topological overlap among genes, with stronger correlations indicated by darker colors. **(D)** Module–trait relationship heatmap displaying the correlation between each module and clinical traits (sepsis vs. control). The “blue” module exhibited the strongest correlation with sepsis (r = −0.58, p = 2e–73). **(E)** Scatter plot showing the positive correlation between module membership and gene significance for sepsis in the blue module (r = 0.72, p = 1.5e–94), suggesting that genes in this module are highly associated with sepsis status.

Supplementary Figure 3 Model performance and evaluation of machine learning algorithms. **(A)** Feature importance ranking of the top 10 genes contributing to model construction. **(B)** Reverse cumulative distribution plots of residuals across four algorithms (RF, SVM, XGB, and GLM), illustrating model stability. **(C–E)** Calibration curve, decision curve analysis, and nomogram of the GLM model. **(F–H)** Calibration curve, decision curve analysis, and nomogram of the SVM model. **(I–K)** Calibration curve, decision curve analysis, and nomogram of the XGB model.

Supplementary Figure 4 Expression and survival analysis of hub genes in the validation cohort. **(A–D)** Violin plots showing the expression levels of hub genes CTSD, ICAM2, SLC22A4, and SNX3 between sepsis and control groups. **(E–H)** K–M survival curves indicating that the expression levels of these genes were not significantly associated with survival outcomes in sepsis patients.

Supplementary Figure 5 The flow chart of the study. Based on the GSE65282 dataset, differentially expressed genes (DEGs) were identified and analyzed for immune cell infiltration using the CIBERSORT algorithm. Consensus clustering was then performed to define subtypes, followed by WGCNA for both cluster-specific and disease-wide analyses. The intersection genes were obtained through Venn analysis and used to construct diagnostic models via machine learning. The model was further validated in external datasets and experimentally confirmed in a CLP model.

## References

[1] Cecconi M Evans L Levy M Rhodes A . Sepsis and septic shock. Lancet. (2018) 392:75–87. doi: 10.1016/S0140-6736(18)30696-2, PMID: 29937192

[2] Schlapbach LJ Watson RS Sorce LR Argent AC Menon K Hall MW . International consensus criteria for pediatric sepsis and septic shock. JAMA. (2024) 331:665–74. doi: 10.1001/jama.2024.0179, PMID: 38245889 PMC10900966

[3] Singer M Deutschman CS Seymour CW Shankar-Hari M Annane D Bauer M . the third international consensus definitions for sepsis and septic shock (Sepsis-3). JAMA. (2016) 315:801–10. doi: 10.1001/jama.2016.0287, PMID: 26903338 PMC4968574

[4] Markwart R Saito H Harder T Tomczyk S Cassini A Fleischmann-Struzek C . Epidemiology and burden of sepsis acquired in hospitals and intensive care units: a systematic review and meta-analysis. Intensive Care Med. (2020) 46:1536–51. doi: 10.1007/s00134-020-06106-2, PMID: 32591853 PMC7381455

[5] Rudd KE Johnson SC Agesa KM Shackelford KA Tsoi D Kievlan DR . Global, regional, and national sepsis incidence and mortality, 1990-2017: analysis for the Global Burden of Disease Study. Lancet. (2020) 395:200–11. doi: 10.1016/S0140-6736(19)32989-7, PMID: 31954465 PMC6970225

[6] Rhodes A Evans LE Alhazzani W Levy MM Antonelli M Ferrer R . Surviving sepsis campaign: international guidelines for management of sepsis and septic shock: 2016. Intensive Care Med. (2017) 43:304–77. doi: 10.1007/s00134-017-4683-6, PMID: 28101605

[7] Walker MK Strich JR . In sepsis, continuous and intermittent infusion of beta-lactam antibiotics did not differ for mortality at 90 d. Ann Intern Med. (2024) 177:JC102. doi: 10.7326/ANNALS-24-01752-JC, PMID: 39222506

[8] Freund Y Cancella de Abreu M Lebal S Rousseau A Lafon T Yordanov Y . Effect of the 1-h bundle on mortality in patients with suspected sepsis in the emergency department: a stepped wedge cluster randomized clinical trial. Intensive Care Med. (2024) 50:1086–95. doi: 10.1007/s00134-024-07509-1, PMID: 38913098

[9] Gotts JE Matthay MA . Sepsis: pathophysiology and clinical management. BMJ. (2016) 353:i1585. doi: 10.1136/bmj.i1585, PMID: 27217054

[10] Liu D Huang SY Sun JH Zhang HC Cai QL Gao C . Sepsis-induced immunosuppression: mechanisms, diagnosis and current treatment options. Mil Med Res. (2022) 9:56. doi: 10.1186/s40779-022-00422-y, PMID: 36209190 PMC9547753

[11] Mayers JR Varon J Zhou RR Daniel-Ivad M Beaulieu C Bhosle A . A metabolomics pipeline highlights microbial metabolism in bloodstream infections. Cell. (2024) 187:4095–4112.e4021. doi: 10.1016/j.cell.2024.05.035, PMID: 38885650 PMC11283678

[12] Barichello T Generoso JS Singer M Dal-Pizzol F . Biomarkers for sepsis: more than just fever and leukocytosis-a narrative review. Crit Care. (2022) 26:14. doi: 10.1186/s13054-021-03862-5, PMID: 34991675 PMC8740483

[13] Seymour CW Kennedy JN Wang S Chang CH Elliott CF Xu Z . Derivation, validation, and potential treatment implications of novel clinical phenotypes for sepsis. JAMA. (2019) 321:2003–17. doi: 10.1001/jama.2019.5791, PMID: 31104070 PMC6537818

[14] Tang D Chen X Kroemer G . Cuproptosis: a copper-triggered modality of mitochondrial cell death. Cell Res. (2022) 32:417–8. doi: 10.1038/s41422-022-00653-7, PMID: 35354936 PMC9061796

[15] Tsvetkov P Coy S Petrova B Dreishpoon M Verma A Abdusamad M . Copper induces cell death by targeting lipoylated TCA cycle proteins. Science. (2022) 375:1254–61. doi: 10.1126/science.abf0529, PMID: 35298263 PMC9273333

[16] Zhang Y Zhou Q Lu L Su Y Shi W Zhang H . Copper induces cognitive impairment in mice via modulation of cuproptosis and CREB signaling. Nutrients. (2023) 15:972. doi: 10.3390/nu15040972, PMID: 36839332 PMC9958748

[17] Wang D Tian Z Zhang P Zhen L Meng Q Sun B . The molecular mechanisms of cuproptosis and its relevance to cardiovascular disease. BioMed Pharmacother. (2023) 163:114830. doi: 10.1016/j.biopha.2023.114830, PMID: 37150036

[18] Springer C Humayun D Skouta R . Cuproptosis: unraveling the mechanisms of copper-induced cell death and its implication in cancer therapy. Cancers (Basel). (2024) 16:647. doi: 10.3390/cancers16030647, PMID: 38339398 PMC10854864

[19] Qin Y Liu Y Xiang X Long X Chen Z Huang X . Cuproptosis correlates with immunosuppressive tumor microenvironment based on pan-cancer multiomics and single-cell sequencing analysis. Mol Cancer. (2023) 22:59. doi: 10.1186/s12943-023-01752-8, PMID: 36959665 PMC10037895

[20] Liao Y Wang D Gu C Wang X Zhu S Zheng Z . A cuproptosis nanocapsule for cancer radiotherapy. Nat Nanotechnol. (2024) 19:1892–902. doi: 10.1038/s41565-024-01784-1, PMID: 39300223

[21] Zhou M Aziz M Yen HT Ma G Murao A Wang P . Extracellular CIRP dysregulates macrophage bacterial phagocytosis in sepsis. Cell Mol Immunol. (2023) 20:80–93. doi: 10.1038/s41423-022-00961-3, PMID: 36471113 PMC9794804

[22] Zhang W Liu Y Liao Y Zhu C Zou Z . GPX4, ferroptosis, and diseases. BioMed Pharmacother. (2024) 174:116512. doi: 10.1016/j.biopha.2024.116512, PMID: 38574617

[23] An S Yao Y Hu H Wu J Li J Li L . PDHA1 hyperacetylation-mediated lactate overproduction promotes sepsis-induced acute kidney injury via Fis1 lactylation. Cell Death Dis. (2023) 14:457. doi: 10.1038/s41419-023-05952-4, PMID: 37479690 PMC10362039

[24] Wu D Spencer CB Ortoga L Zhang H Miao C . Histone lactylation-regulated METTL3 promotes ferroptosis via m6A-modification on ACSL4 in sepsis-associated lung injury. Redox Biol. (2024) 74:103194. doi: 10.1016/j.redox.2024.103194, PMID: 38852200 PMC11219935

[25] Xue Q Kang R Klionsky DJ Tang D Liu J Chen X . Copper metabolism in cell death and autophagy. Autophagy. (2023) 19:2175–95. doi: 10.1080/15548627.2023.2200554, PMID: 37055935 PMC10351475

[26] Lee SG Woo SM Seo SU Lee CH Baek MC Jang SH . Cathepsin D promotes polarization of tumor-associated macrophages and metastasis through TGFBI-CCL20 signaling. Exp Mol Med. (2024) 56:383–94. doi: 10.1038/s12276-024-01163-9, PMID: 38297161 PMC10907383

[27] Fleuren LM Klausch TLT Zwager CL Schoonmade LJ Guo T Roggeveen LF . Machine learning for the prediction of sepsis: a systematic review and meta-analysis of diagnostic test accuracy. Intensive Care Med. (2020) 46:383–400. doi: 10.1007/s00134-019-05872-y, PMID: 31965266 PMC7067741

[28] Theodosiou AA Read RC . Artificial intelligence, machine learning and deep learning: Potential resources for the infection clinician. J Infect. (2023) 87:287–94. doi: 10.1016/j.jinf.2023.07.006, PMID: 37468046

[29] Bomrah S Uddin M Upadhyay U Komorowski M Priya J Dhar E . A scoping review of machine learning for sepsis prediction- feature engineering strategies and model performance: a step towards explainability. Crit Care. (2024) 28:180. doi: 10.1186/s13054-024-04948-6, PMID: 38802973 PMC11131234

[30] Guo X Li H Meng X Zhao Z Zhang R Wang L . CD8 + T-cell number and function are altered by Shkbp1 knockout mediated suppression of tumor growth in mice. Mol Immunol. (2023) 160:32–43. doi: 10.1016/j.molimm.2023.06.004, PMID: 37343421

[31] Halai K Whiteford J Ma B Nourshargh S Woodfin A . ICAM-2 facilitates luminal interactions between neutrophils and endothelial cells. vivo. J Cell Sci. (2014) 127:620–9. doi: 10.1242/jcs.137463, PMID: 24317296 PMC4007766

[32] Yu W Hu Y Liu Z Guo K Ma D Peng M . Sorting nexin 3 exacerbates doxorubicin-induced cardiomyopathy via regulation of TFRC-dependent ferroptosis. Acta Pharm Sin B. (2023) 13:4875–92. doi: 10.1016/j.apsb.2023.08.016, PMID: 38045054 PMC10692393

[33] Pochini L Galluccio M Console L Scalise M Eberini I Indiveri C . Inflammation and organic cation transporters novel (OCTNs). Biomolecules. (2024) 14:392. doi: 10.3390/biom14040392, PMID: 38672410 PMC11048549

[34] Dikic I . CIN85/CMS family of adaptor molecules. FEBS Lett. (2002) 529:110–5. doi: 10.1016/s0014-5793(02)03188-5, PMID: 12354621

[35] Soubeyran P Kowanetz K Szymkiewicz I Langdon WY Dikic I . Cbl-CIN85-endophilin complex mediates ligand-induced downregulation of EGF receptors. Nature. (2002) 416:183–7. doi: 10.1038/416183a, PMID: 11894095

[36] Johnson RI Seppa MJ Cagan RL . The Drosophila CD2AP/CIN85 orthologue Cindr regulates junctions and cytoskeleton dynamics during tissue patterning. J Cell Biol. (2008) 180:1191–204. doi: 10.1083/jcb.200706108, PMID: 18362180 PMC2290846

[37] Kuhn J Wong LE Pirkuliyeva S Schulz K Schwiegk C Funfgeld KG . The adaptor protein CIN85 assembles intracellular signaling clusters for B cell activation. Sci Signal. (2016) 9:ra66. doi: 10.1126/scisignal.aad6275, PMID: 27353366

[38] Cancer Genome Atlas Research N Albert Einstein College of M Analytical Biological S Barretos Cancer H Baylor College of M Beckman Research Institute of City of H . Integrated genomic and molecular characterization of cervical cancer. Nature. (2017) 543:378–84. doi: 10.1038/nature21386, PMID: 28112728 PMC5354998

[39] Gyorffy B . Discovery and ranking of the most robust prognostic biomarkers in serous ovarian cancer. Geroscience. (2023) 45:1889–98. doi: 10.1007/s11357-023-00742-4, PMID: 36856946 PMC10400493

[40] Liu Q Li H Yang M Mei Y Niu T Zhou Z . Suppression of tumor growth and metastasis in Shkbp1 knockout mice. Cancer Gene Ther. (2022) 29:709–21. doi: 10.1038/s41417-021-00349-x, PMID: 34112919

[41] Wang J Zhang Y Ma T Wang T Wen P Song W . Screening crucial lncRNAs and genes in osteoarthritis by integrated analysis. Adv Rheumatol. (2023) 63:7. doi: 10.1186/s42358-023-00288-1, PMID: 36849988

