# Supplementary figures and images for: SHKBP1 is a target for sepsis: evidence from WGCNA and multiple machine learning algorithms
Source: Front Immunol. 2025 Dec 8;16:1709188. doi: 10.3389/fimmu.2025.1709188 (PMC12722824; doi:10.3389/fimmu.2025.1709188)

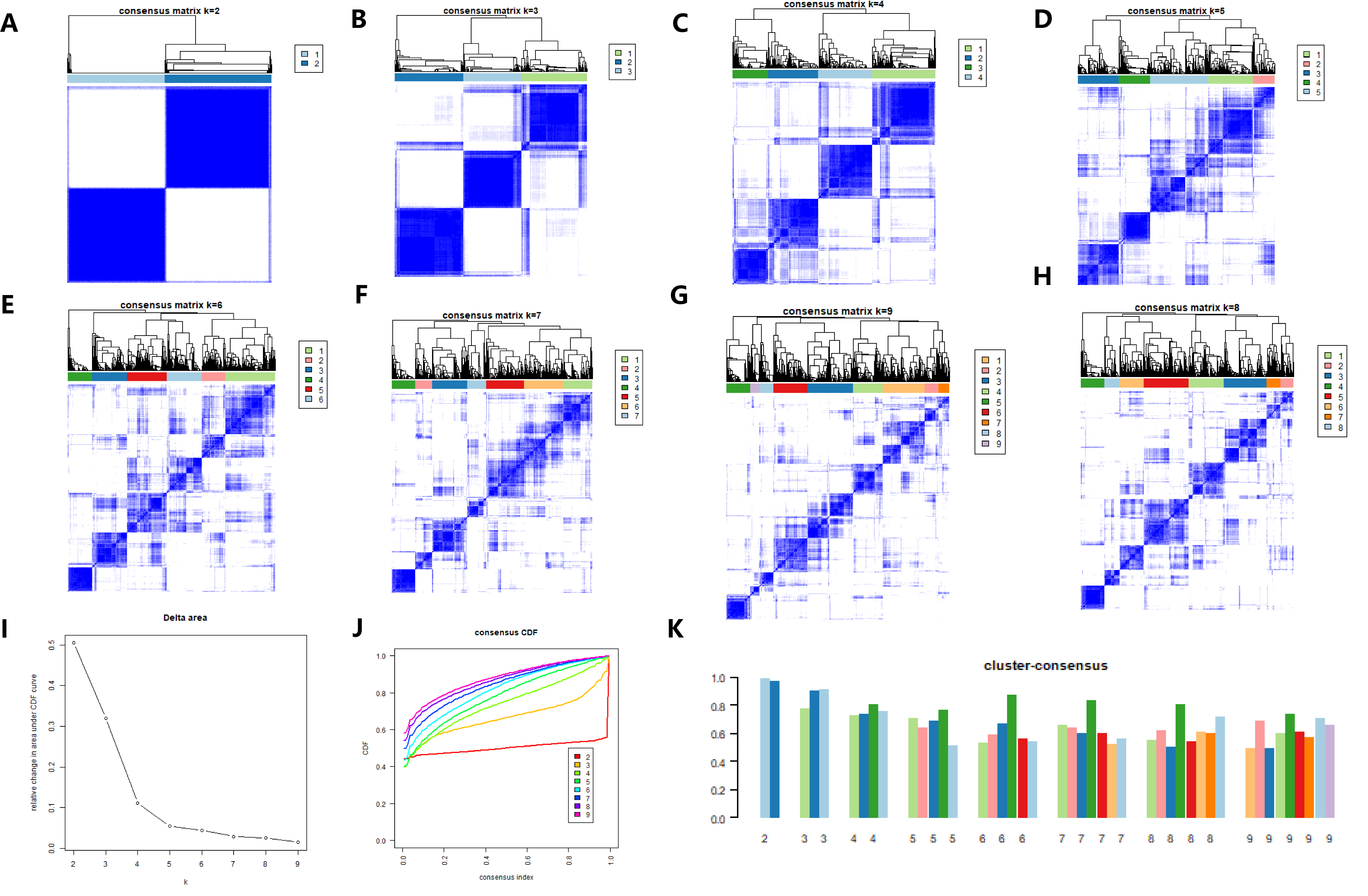

Supplement: Supplementary Figure 1 — Consensus clustering analysis in sepsis samples. (A–H) Consensus matrices for different cluster numbers (k = 2–9), where the intensity of blue indicates the consensus within clusters. (I) Delta area plot showing the relative change in the area under the cumulative distribution function (CDF) curve for each k value. (J) CDF curves of consensus scores across different cluster numbers. (K) Histogram of cluster consensus scores, demonstrating optimal clustering stability when k=2. [file Image1.tif]

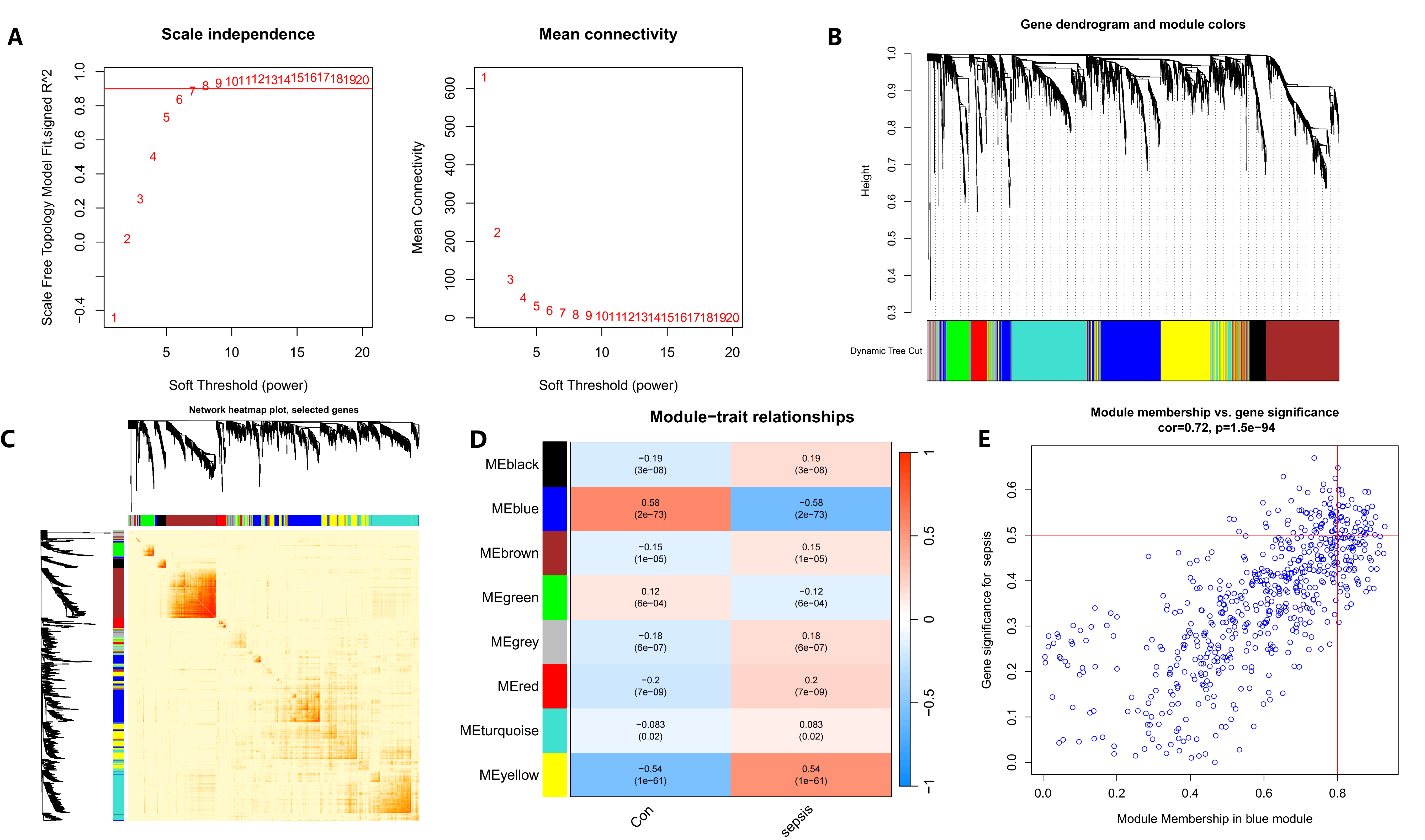

Supplement: Supplementary Figure 2 — WGCNA of sepsis and healthy control samples. (A) Analysis of network topology across a range of soft-thresholding powers. The scale-free topology fit index (left) and mean connectivity (right) were used to determine the optimal power for network construction. (B) Gene dendrogram showing hierarchical clustering of genes into distinct co-expression modules, each represented by a different color. (C) Heatmap visualizing the topological overlap among genes, with stronger correlations indicated by darker colors. (D) Module–trait relationship heatmap displaying the correlation between each module and clinical traits (sepsis vs. control). The “blue” module exhibited the strongest correlation with sepsis (r = −0.58, p = 2e–73). (E) Scatter plot showing the positive correlation between module membership and gene significance for sepsis in the blue module (r = 0.72, p = 1.5e–94), suggesting that genes in this module are highly associated with sepsis status. [file Image2.tif]

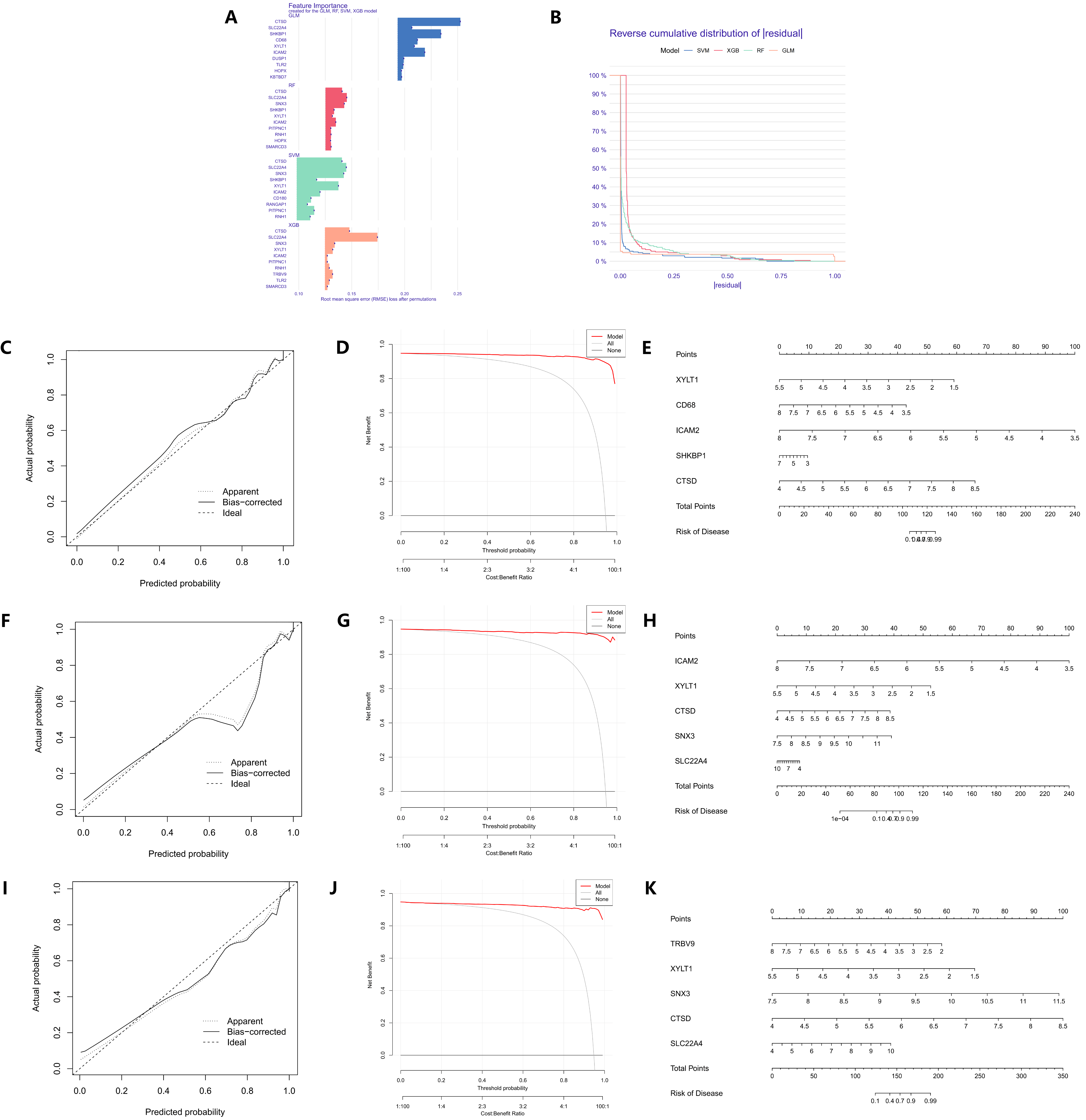

Supplement: Supplementary Figure 3 — Model performance and evaluation of machine learning algorithms. (A) Feature importance ranking of the top 10 genes contributing to model construction. (B) Reverse cumulative distribution plots of residuals across four algorithms (RF, SVM, XGB, and GLM), illustrating model stability. (C–E) Calibration curve, decision curve analysis, and nomogram of the GLM model. (F–H) Calibration curve, decision curve analysis, and nomogram of the SVM model. (I–K) Calibration curve, decision curve analysis, and nomogram of the XGB model. [file Image3.tif]

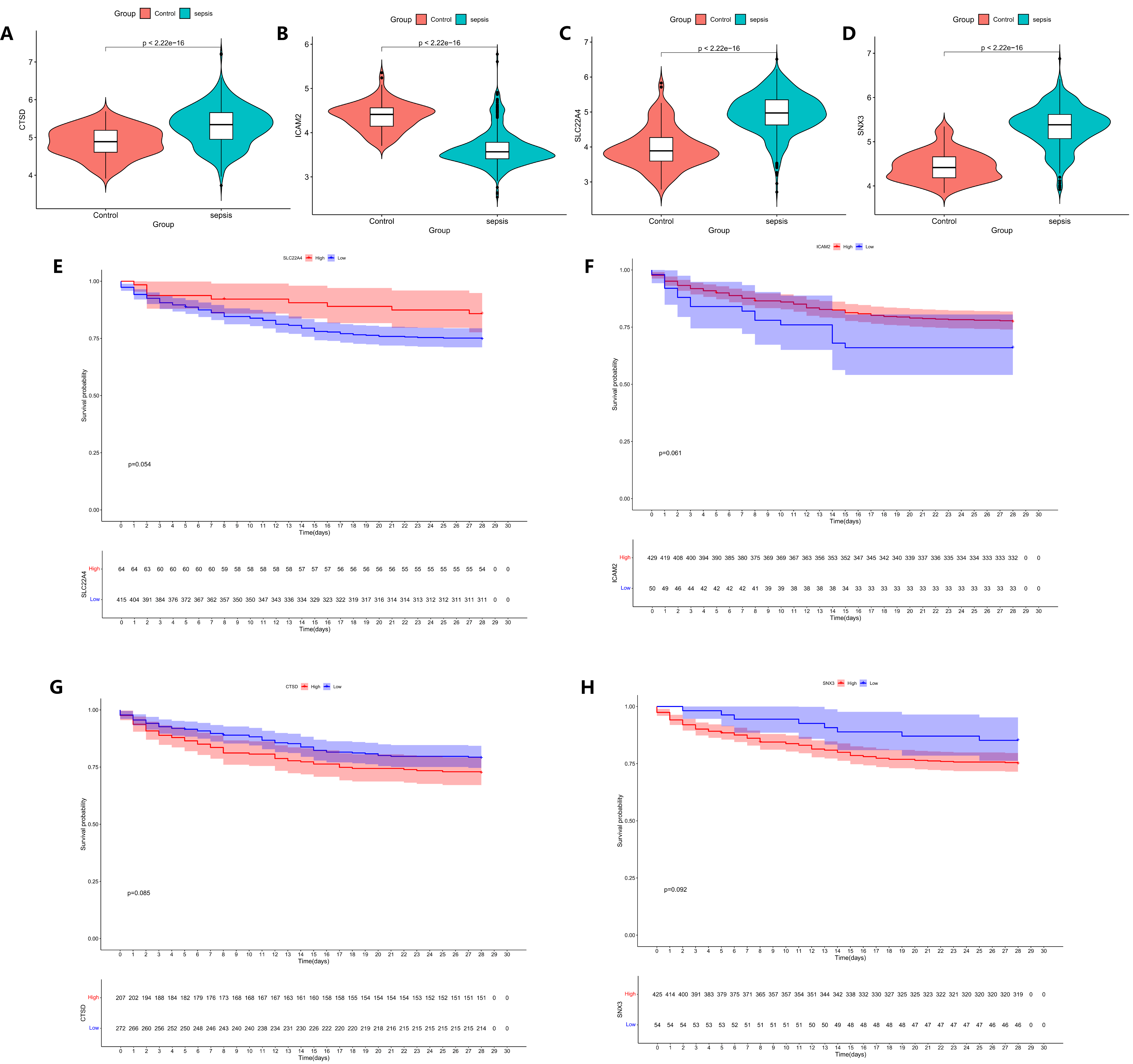

Supplement: Supplementary Figure 4 — Expression and survival analysis of hub genes in the validation cohort. (A–D) Violin plots showing the expression levels of hub genes CTSD, ICAM2, SLC22A4, and SNX3 between sepsis and control groups. (E–H) K–M survival curves indicating that the expression levels of these genes were not significantly associated with survival outcomes in sepsis patients. [file Image4.tif]

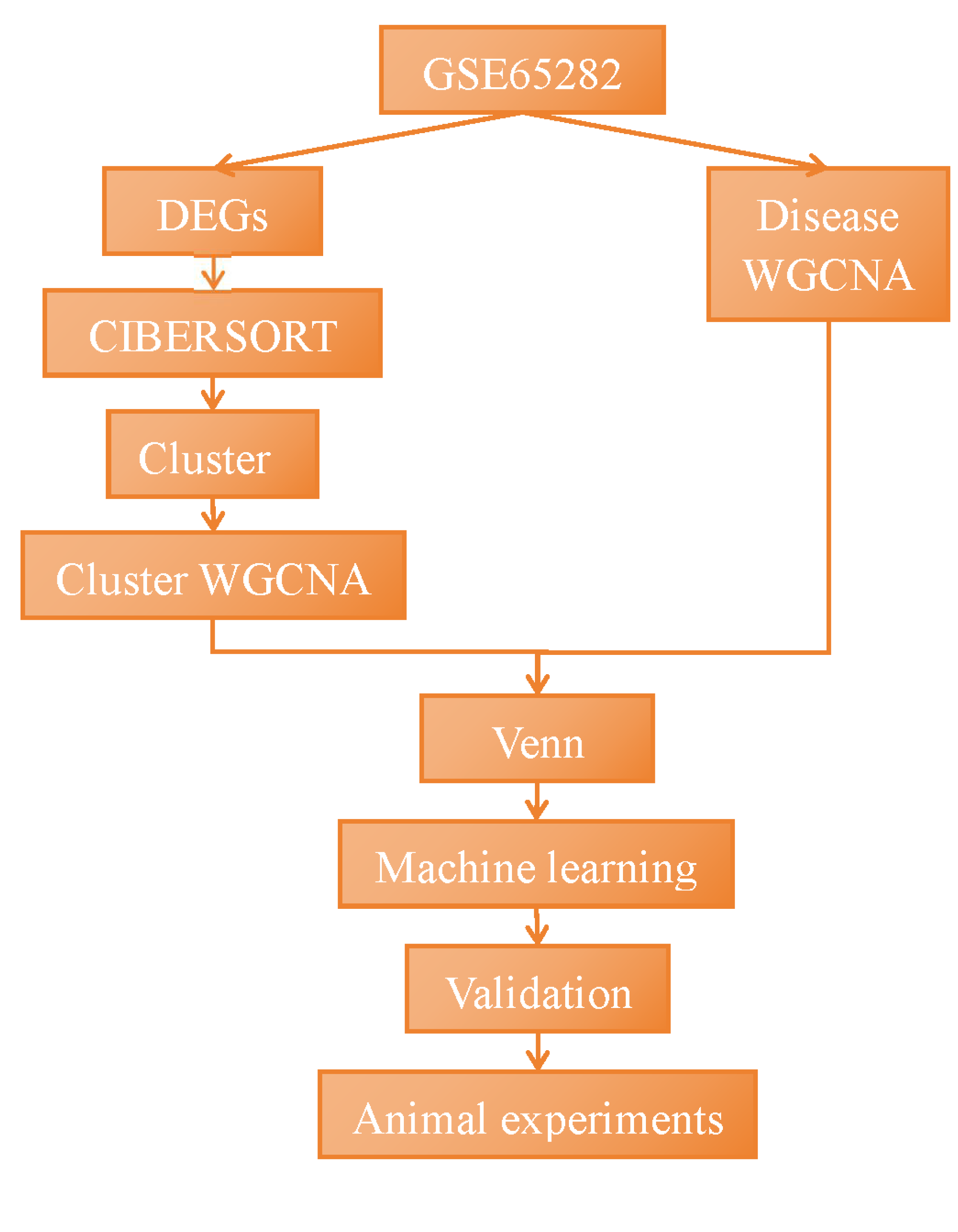

Supplement: Supplementary Figure 5 — The flow chart of the study. Based on the GSE65282 dataset, differentially expressed genes (DEGs) were identified and analyzed for immune cell infiltration using the CIBERSORT algorithm. Consensus clustering was then performed to define subtypes, followed by WGCNA for both cluster-specific and disease-wide analyses. The intersection genes were obtained through Venn analysis and used to construct diagnostic models via machine learning. The model was further validated in external datasets and experimentally confirmed in a CLP model. [file Image5.tif]
